# Shifting Stakes: Understanding the Dynamic Roles of Individuals and Organizations in Social Media Protests

**DOI:** 10.1371/journal.pone.0165387

**Published:** 2016-10-24

**Authors:** Emma S. Spiro, Andrés Monroy-Hernández

**Affiliations:** 1 Information School & Department of Sociology, University of Washington, Seattle, WA, United States of America; 2 Microsoft Research, Redmond, WA, United States of America; Universitat Rovira i Virgili, SPAIN

## Abstract

In this paper we examine two protests characterized by substantial social media presence and distributed participation frameworks via two core questions: what roles did organizations and individuals play, and how did participants’ social interactions change over the course of the protests? To answer these questions, we analyzed a large Twitter activity dataset for the #YoSoy132 student uprising in Mexico and Brazil’s “bus rebellion.” Results indicate that individuals initially took prominence at the protests but faded in importance as the movements dwindled and organizations took over. Regarding the dynamics and structure of the interactions, we found that key time points with unique social structures often map to exogenous events such as coordinated protests in physical locations. Our results have important consequences for the visibility of such social movements and their ability to attract continued participation by individuals and organizations.

## Introduction

Two waves of large protests took place in Mexico during May of 2012 and in Brazil during May of 2013. Triggered by seemingly small incidents (a politician’s dismissal of hecklers in Mexico, and bus fare increases in Brazil), these protests grew and later encompassed much larger and more intricate societal problems such as media monopolies, corruption, and income inequality. Both protests gained national and international attention for their ability to mobilize people online and offline. Also, their prominent social media presence prompted journalists and scholars to point out their similarity to the “Arab Spring,” “Occupy” and other hashtagged uprisings [1–3]. Despite their size and notoriety, however, and unlike their Arab counterparts, the Mexican and Brazilian protests both dwindled months later, leading some to question the staying power and effectiveness of these new, social media-focused forms of social and political movements.

Even before social media became popular, researchers had begun to examine the relationship between social movements and networked technologies. For instance, as early as 1997, scholars examined the use of the internet by the Zapatista movement in Mexico and argued that the movement was able to overcome the traditional geographic constraints of access to media thanks to the “constant and reciprocal connections between cyberspace and other social spaces” [4]. Similarly, others have noted the emergence of new forms of political dissent mediated by technologies like SMS in the“Pasalo” movement in Spain [5], and by modern tools such as Facebook and Twitter during the Occupy Wall Street movement in the United States [6, 7] and the Arab Spring in Egypt [8–10].

The exact role of social media in sparking, organizing, and sustaining political protests has been the topic of much debate [11]: some people argue that social media is a key factor in the emergence of recent uprisings [10, 12], while others have voiced skepticism due to social media’s apparent reliance on weak links for recruitment in joining protests [13, 14]. What is clear is that social media does play a role during these protests. Part of the challenge, however, in characterizing this role is that social media are used both by institutions and individuals. The interactions within and among these different types of actors have not been fully examined; we aim to fill part of that gap in this paper.

In this work, we examine the communication structures, and the roles of individuals and organizations in these uprisings, to further our understanding of these new forms of political dissent. New theories of connective action posit that these networks and the roles that actors occupy are very different from those formed during traditional institutionally-driven social movements, partially due to the personalization of political action and the affordances of social media. In particular, we examine differences in the activity patterns and embeddedness over time for these two different types of actors on social media.

## Personalized Politics and Connective Action

While debate about the specific role of social media and networked technologies will continue, it is clear from recent events that platforms like Twitter will be used to exchange information, share stories, and interact with other participants during political protests. The affordances of these tools match protestors’ need or desire to share personalized stories. This type of behavior is identified by [15] as the “linchpin” of what the authors call *connective action*—a growing phenomenon in which unaffiliated citizens coalesce around undefined movements [16]. In this context, individuals are able to personalize their participation and interaction with the protest itself, leading to the development of content that is subsequently distributed widely across diverse social networks.

One of the core distinctions that Bennett and colleagues make in separating new connective action frames from more traditional collective action frames concerns the role of traditional institutions or organizations in setting and sustaining the movements’ aims and objectives. Indeed, scholars have noted that new forms of social media enabled protests seem “to operate with surprisingly light involvement from conventional organizations” [15]. More traditional social movements, on the other hand, rely on the coordination of action by organizations. Newer forms of political protests operating via digitally enabled action networks are thought to have only loose or little involvement with traditional organizations. Despite this hypothesized distinguishing feature, little is known about the role that organizations play in online communication and coordination longitudinally over the lifetime of “hashtagged” political protests.

This is not to say that researchers have not investigated the role of social media in facilitating coordination and information exchange during social protests. In one recent study, it was demonstrated that mass protest events are associated with observed coordination activity on Twitter [10]; this work goes further to argue that observed coordination is not the result of traditional actors such as media or elites, but rather distributed social networks. Others have likewise explored how the structure of networked social movements can present both opportunities and barriers to information diffusion, mobilization and opinion change [17, 18]. However, despite these important advances in understanding the role and possible consequences of utilizing social media for social movements, many open questions remain.

Our work addresses these gaps, focusing specifically on the distinct roles that individuals and organizations play in digitally enabled networks over time. In particular we address the following research questions: *(1) what are the roles of organizations and individuals over the course of the life of the protests? and (2) how do interactions among participating actors change as protests grow and eventually dwindle?* In comparison to other work in this area, we focus specifically on two social protests where we can observe protest of their lifetime.

## Background

### Mexican Student Uprising

Two months before the 2012 Mexican presidential election, the “Yo Soy 132” uprising (“I am 132” in Spanish) started when protesters gathered at a visit from (then) presidential candidate Enrique Peña Nieto (EPN) at the Universidad Iberoamericana in Mexico City. Shortly after fleeing the university due to the protests, the candidate and his party’s leadership accused the protesters of intolerance, and questioned their affiliation and true motives. The claim was that they were “too old to be students” and that they had been manipulated by EPN’s political opponents. The next day, the reporting of the incident by several news outlets was in stark contrast to the students’ version of events. For example, many of the students particularly resented a headline by a major newspaper that reported how the candidate had left the university “triumphant” in spite of an “orchestrated boycott attempt.”

Angered by the accusations and the unfair media coverage, the students uploaded a video to YouTube titled “131 students of Ibero respond.” The video showed one student after another, stating their name while holding their student ID card, and denying being paid by anyone to protest that day. After the video went viral, people started to show their support for the protests with the Twitter hashtag #YoSoy132 or I am 132. The protests spread to several cities in Mexico and throughout the Mexican diaspora around the world.

The protest’s demands were primarily centered around the “democratization of the media,” after what was perceived by many to be a campaign driven by large media corporations attempting to manufacture EPN’s candidacy. Later the demands broadened to include most of the challenges confronting a large portion of Mexican society. Eventually EPN was officially elected president, and took office on December 1, 2012, a day marked not only by violent protests that sent several people to jail, but also the final large-scale protests of the YoSoy132 movement. Table 1 contains a timeline of major events during the Mexican case.

**Table 1:** Timeline of major events during the Mexican YoSoy132 protest.

May 11	Presidential EPN candidate heckled at university
May 12	News media coverage upsets students
May 14	Students post famous YouTube video that gave name to the protests
June 9	Massive protests on the streets of Mexico City
June 15	Concert organized by the protesters in the main plaza of Mexico City
June 19	Presidential debate organized by the protestors
July 1	Presidential election
July 27	Protesters occupy offices of Televisa, major media conglomerate
December 1	EPN takes office

### Brazilian “Bus Rebellion”

More than a million Brazilians joined protests in over 100 cities throughout Brazil in June 2013. The protests were motivated by the increase in bus fares and quickly earned the name of the “Revolta do Busão” (bus rebellion). The protesters went out on the streets not only in major Brazilian cities, but also abroad. The movement was seen as the largest in the last two decades [19] and forced state and local authorities to engage in dialogue with the protesters.

The first demonstrations in June took place in São Paulo, organized by Movimento Passe Livre (MPL). The group used social networking sites and mobile devices to coordinate events. The protests were sparked by the increase of the bus fare in the state capital, from R$ 3.00 to R$ 3.20. After the first two weeks of intense protests in June, the governments of São Paulo and other cities dropped the fare increase and announced the reduced bus rates. But protest leaders were not satisfied with the government’s solution and demonstrations continued in the streets, demanding better-quality public transportation. The movement was characterized by non-partisanship and was led by young college students. Protesters were angry about corruption, inequality, and the high cost of hosting the upcoming World Cup and Olympic games. They were mostly demanding that the investments given to the sport events be equaled by investments in public services.

In the early protests, the vandalized buses, banks, subway stations, and assets aroused the antipathy of the general public. All this changed on June 13th, when military police harshly repressed the demonstration at Avenida Paulista, the financial center of the city, injuring civilians and journalists. That violence echoed outside Brazil, causing disapproval by organizations such as Amnesty International. The first big protest happened on June 17, when more than 270,000 protesters took the streets in more than 30 cities in Brazil. Thousands of protesters attended several marches that despite the occasional vandalism won the support of the general public and political leaders.

Police repression served as fuel for the protesters. In the following days, the protests gained the attention and support of international movements and the wave of protests had spread to the main Brazilian cities. Dozens of other protests in 27 countries were organized to support the Brazilian protesters. Images of the protests in Brazil were also carried by the international press. Stadiums where the Brazilian national team was playing in the Confederations Cup games served as stages for the protesters. As Brazil defeated Spain to win the Confederations Cup final, police clashed with protesters near Maracanã stadium for the second time in two weeks. Table 2 contains a timeline of major events during the Brazilian case.

**Table 2:** Timeline of major events during the Brazilian bus rebellion protest.

January 14	Mayor announces bus fare increase
June 16	Massive protest in Rio de Janeiro
June 18	Protesters take to the streets of several cities
June 19	City governments rescind bus fare increase
June 21	President issues statement to address protesters

## Data Collection

In support of this research we collected data from the popular microblogging service Twitter. Data was accessed via the Twitter Firehose application programming interface (API), made available to our research team through a company agreement with Twitter. This Twitter API access point returns all public tweets based on specific keyword-based search queries. For each case study we identify a list of movement-related hashtags based on trending topics, news media, and a snowball sample of Twitter message themselves. Hashtag-based sampling it just one method for capturing event-related data. It has been used by many researchers, and has also been criticized. We believe it provided a good sample for this case, however we acknowledge the need for further work to evaluate these types of sampling strategies. Since these “hashtagged social protests” are notably and publicly tied to these tags, this method for data collection also provides a view of the event from the perspective of a newcomer searching the social media space with that tag. This is a common approach used in social media research to identify content surrounding events of interest [20–22]. A list of the primary hashtags used is found in Table 3; a full list is available upon request, however, those listed account for the large majority of data used in this study. For the Mexican protest, one hashtag was by far the most prominent. We accessed the Twitter Firehose API to collect all tweets containing each set of hashtags for the observation periods listed in Table 4. We collected a total of 2,984,193 tweets from the Mexican protest and 3,877,181 from the Brazilian protest. These methods adhered to the terms and conditions of the Twitter API as well as the licensing of these data. While Twitter data are publicly available, user accounts may contain personally identifying information. To maintain participants’ privacy, we do not identify or report on individual accounts.

**Table 3:** List of example hashtags used in data collection for each social protest.

Mexican Protest	Brazilian Protest
#yosoy132	#foracut
	#abaixoredeglobo
	#foradilma
	#forapt
	#pec37

**Table 4:** Descriptive statistics for the two social protest-related online communication datasets.

	Mexican Protest	Brazilian Protest
Tweets	2,984,193	3,877,181
Users	470,485	957,965
Start Date	2012-05-15	2013-05-01
End Date	2012-12-31	2013-07-29
Avg. Hourly Rate	541	1,801
Med. Hourly Rate	194	138
Max. Hourly Rate	32,061	243,351
Avg. Tweet Per User	6	4
Med. Tweet Per User	2	2
Max. Tweets Per User	4,480	23,283

Using these larger datasets as a starting point, we focus on multiple aspects of the data in the subsequent analysis. First we consider the overall pattern of communication, exploring both temporal and content features of the data for each protest. Second, we look specifically at Twitter-based interaction among active actors (which we define and discuss in more detail below). We consider active users because a large majority of users only appear in the dataset once; it is those users who demonstrate repeated action that participate in the movement over time. We take a sample of these highly active, highly embedded users and perform a manual coding task to determine whether the account represented an organization or an individual.

### Interaction Networks

There are many different ways to define social interaction among participants on social media platforms; Twitter for example has following relationships, direct messaging relationships, retweeting activity, etc. [23]. We define interaction as any kind of mention of another user, encompassing public directed messages, mentions, and retweeting. In other words, interaction on Twitter for the purposes of the subsequent analysis indicates direct awareness of another account. This awareness of other users could be gained through exposure via searches of the public timeline or through more durable following relationships. In both cases, we believe this active awareness is a stronger type of interaction than more general passive following relationships, which could be formed and then forgotten over time.

Using the dataset described we construct a social network of interaction among individuals who participate in the movement-related conversation. Because individual posting rates are highly skewed—a large proportion of actors post very few event-related messages—we chose to focus on users who are active over time. For the case of Mexico this is users who posted at least 86 times over the observation period of 7 months. For Brazil we considered the population of users who posted at least 37 messages over two months. In both cases this is the top 1% of users according to the activity distribution. This sampling gives a total of 4,705 and 9,319 users in the population of interest for Mexico and Brazil respectively. In the subsequent analysis we discuss the induced subgraph of activity among these users, i.e. only the interactions observed between this set of highly active users.

### Coding Participants Types

Many of the previously discussed theories of personalized politics and online social movements suggest that the roles played by individual people and organizations are different, both during the initial “creation” of the protest and as the protests continues over time. To explore these theories, we offer an in depth analysis of a sample of highly active and highly embedded users, looking at how the behaviors of these actors may differ based on the individual/organization dichotomy. This analysis requires that we manually code a sample of Twitter accounts, classifying accounts (i.e. usernames) as associated with individual people or with organizations.

We sample a set of users to code by considering the most central users in the interaction network so as to focus the coding task to a manageable set of accounts. We compute five different social network centrality measures on daily snapshots of the interaction network, as well as the interaction network aggregated over time. Centrality measures used include: total degree, indegree, outdegree, betweenness and eigenvector centrality. In practice, in many cases, these five measures are highly correlated; while the same individual often score highly across all five there are also differences so we chose to use all of them in the sampling procedure. In the case of the daily snapshots of the network we sample the top ten users by each centrality measure. For the aggregate network over time and we sample the top 100 people according to the same centrality indices. This sampling strategy resulted in 977 unique accounts for Mexico and 898 unique user accounts for Brazil.

Three independent coders who were familiar with the region and the protest events were asked to classify the accounts. We provided them with all information available on an individual accounts Twitter profiles. Coders were asked to classify accounts as organizations or individuals. We did not provide a strict definition of each category, but rather chose to rely on the coders’ expert judgement of the boundary between these two types of user accounts. This procedure was designed to elicit a classification that matched user perceptions.

We use Fleiss’ Kappa to assess the reliability of the agreement between our three raters for the binary distinction between individual accounts and organizational accounts. *κ* = 0.799 for the Mexican data and *κ* = 0.696 for the Brazilian data. In general, a *κ* > 0.60 is considered to be substantial agreement between the coders. Reliability for this procedure is very high, even given the lack of formal definitions for the account categories indicating that users’ perceptions of individual versus organizational accounts were consistent; we used majority rule to determine the final code.

## Results

Using the data described above, we explore the temporal, relational, and content features of protest-related messages posted to Twitter. We compare the behavioral and interaction patterns of individual or organizational actors participating in the production, dissemination, and discussion of this content online. In each of the subsequent analysis we address the research questions discussed previously; we aim to characterize observed patterns for these two cases—both similarities and differences between the two cases are discussed.

### Temporal Dynamics

Table 4 contains descriptive statistics for each protest dataset before our sampling strategy is employed. We collected over 2.9 million tweets for the Mexican protests and 3.8 million tweets for the Brazilian protests. These tweets represent the activity of more than 450,000 unique users in the Mexican case, and just under 1 million unique users for the Brazilian case. While a large number of individuals posted at least one movement-related message on Twitter, fewer individuals have repeated participation. For both Mexico and Brazil, more than 50% of unique users posted fewer than 3 messages over the observation period.

Table 4 also demonstrates diversity in activity, seen through average posting behavior. Users posted an average of around 5 messages total over the observation period; the median number of messages is 2 for both cases studied. On the other hand, while most actors participated very little, we did observe some participants who posted thousands of times.

**Fig 1:**
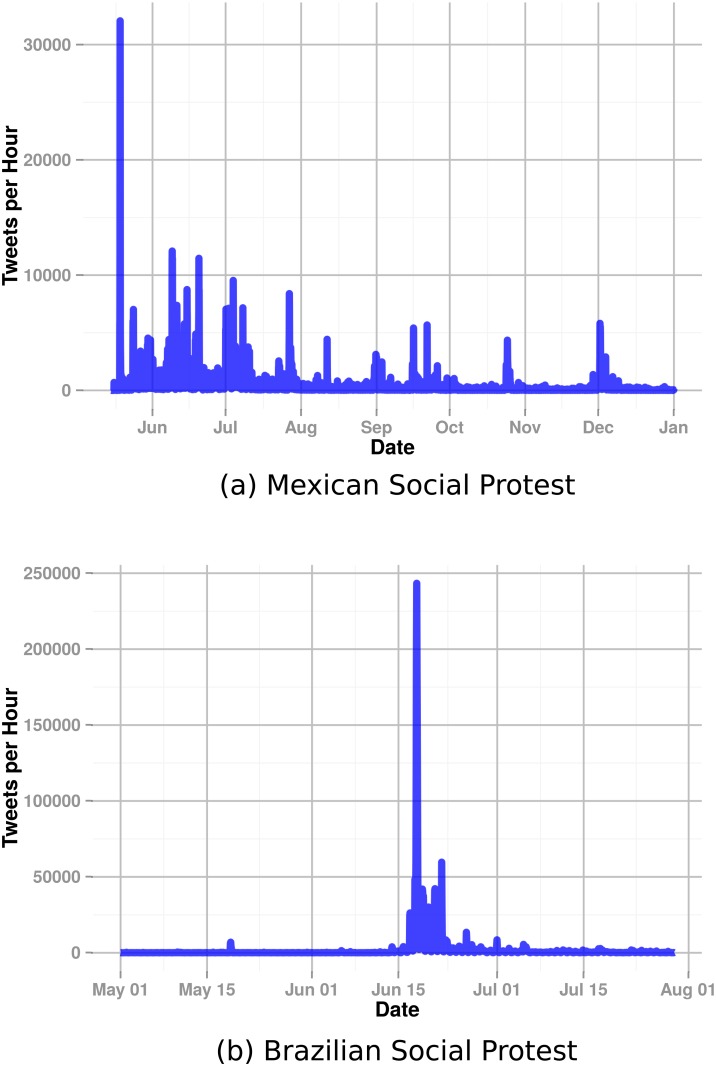
Count of messages (tweets) per hour posted to Twitter for each social protest over time.

Fig 1 shows the time series of hourly Twitter posts for each case study. Clearly, the two cases differ with respect to their overall activity patterns. As this figure demonstrates, the Mexican protest has multiple periods of increased activity, while the Brazilian one has highly concentrated activity surrounding a single peak. One explanation for these two distinct patterns is that after a large demonstration the Brazilian government conceded some of the protesters demands [3], while the Mexican institutions never really addressed the uprising’s demands. These overall temporal dynamics offer a high-level picture of the “life course” of the movement and how it unfolds on Twitter.

### The Content of Communication

Next, we explore specific content features observed in the messages posted. Content in many social media is restricted, Twitter for example only allows messages that are a maximum of 140 characters in length. As a result, users have adopted conventions for adding metadata, bringing messages to the attention of others, and attributing informational content to others. Retweets—messages that were originally posted by another user and are then reposted by someone else—are one example of this type of behavior [23]. 56.1% of tweets collected for the Mexican case and 55.2% of the tweets collected in the Brazilian case are official retweets. Replies are rarely used, approximately 5% in both cases. 47% of Mexican protest-related tweets contained a URL, compared to 35% in the Brazilian case. One interesting difference was in the proportion of tweets that were posted using the Twitter Web interface, rather than a mobile client—39% for the Mexican case and 50% for the Brazilian case.

In both cases, a large portion of communication represents serial transmission of messages posted by others. Platforms such as Twitter have been recognized for their potential to rapidly spread information through mechanisms such as retweets [24]. Direct replies to other users are much less utilized, less than 5% of tweets fall into this category in both cases. Some scholars have similarly noted that Twitter is less a conversation platform than it is a dissemination platform [20].

Furthermore, almost half the tweets in the dataset contain links to other information—both internal (to content in Twittter) and external. Interestingly, if we consider only original content (tweets that are not retweets) we find that 27% of tweets have URLs in them for the Mexican protest data, while this percentage is 16% for the Brazil case. Studies of information flow between social media and other media platforms is an area for future work.

We also investigated the most represented domains for linked URLs. For the Mexican protests, half of the 10 most frequently linked websites were to news media sources, namely: Proceso (political news magazine), Aristegui Noticias (news portal named after a well-known journalist), SinEmbargo MX (online news portal), La Jornada (liberal newspaper from Mexico City), and Milenio (national newspaper). For the Brazilian case, only two news media sources were highly linked, namely, Folha de S. Paulo (newspaper) and Globo (news portal).

In addition to mainstream media, tweets with links to other social media platform were popular. YouTube and Facebook were among the top referenced sites for both the Mexican and the Brazilian protests. However, for Brazil, Instagram and Twitcasting, a live video service, were highly popular, but were completely absent from the Mexican protests. More broadly, 41 of the 100 most frequently tweeted URLs in the Mexican case, and 62 in the Brazilian one, are links to videos and images–often used to broadcast the live or recorded videos of the protests, public announcements, or even celebrities’ support for the movements.

The last feature of the overall activity we consider, before turning to the interaction data, is the geospatial distribution of conversation participants. Many “hashtagged social protests” include participants from around the globe. We explore the top represented timezones in our data and number of unique users posting from each. In both cases we find that the local timezone is among the top two, as might be expected. Also, in the case of the Brazilian protests, messages with the Istanbul, Turkey timezone were common enough to appear among the top. This is particularly interesting given that Turkey had a similar large wave of protests occurring at the same time. Some of the images shared on social media also showed people in Brazil displaying banners in the street in support of their Turkish counterparts.

### Personalized Politics

Our primary focus in this study is to explore the roles played by individuals and organizations in terms of communication surrounding each protest. Recall, as part of the data collection we manually coded a sample of highly active, highly embedded Twitter accounts. Table 5 gives basic statistics of the activity and content posted by these users, comparing organizations with individuals.

**Table 5:** Descriptive statistics of activity by actor role.

	Mexican Protest		Brazilian Protest	
	Individuals	Organizations	Individuals	Organizations
Min. Tweets	4	14	3	3
Mean Tweets	289.963	416.176	182.611	372.87
Median Tweets	202.5	247	109	121
Max. Tweets	2,666	2,555	4,720	23,283
Mean Prop. of Tweets with URL	0.637	0.335	0.522	0.376
Mean Prop. Retweet	0.078	0.065	0.1	0.06
Mean Prop Replies	0.511	0.583	0.341	0.505

Organizations produce a larger number of posts on average per account over time than individuals. There are noticeable outliers—one account posted over 23,000 messages during the Brazilian protest.We find an overall pattern of high proportions of retweets across both actor categories. In some cases over half of the posts from an average user contain links to external information. Individuals tend, on average, to have a higher proportion of retweets than organizations. Replies are rarely used by either actor type. This supports recent work demonstrating that social media platforms such as Twitter are valuable for dissemination and spread of information but don’t facilitate in-depth conversations [20].

**Fig 2:**
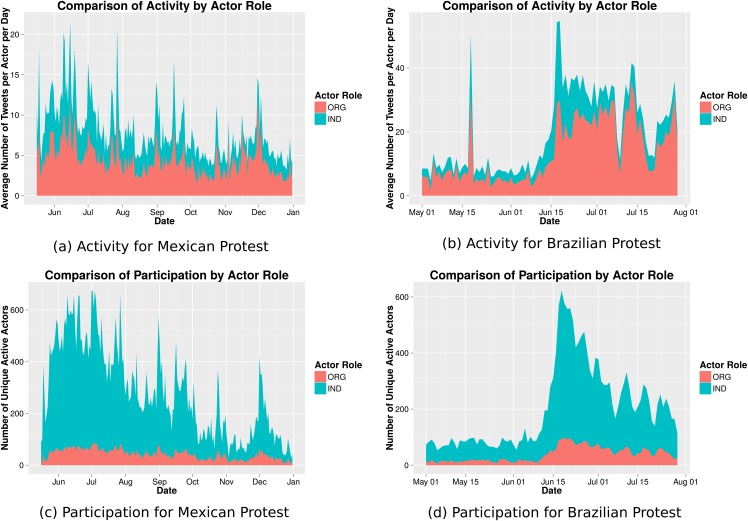
Average Twitter activity (tweets per day) among a sample of highly active, highly embedded users by actor type on top. Participation by actor type for the same set of users over time on bottom.

Fig 2, top panel, shows a comparison of average daily activity for our population of interest. The width of each ribbon in this visualization conveys the average number of messages posted per actor per day, such that the combined height of the colored area shows the total amount of activity observed.

Activity tends to occur in bursts. In some cases, bursts match exogenous events in the offline environment, such as demonstrations, rallies, or protests. For example, we see the biggest spike of activity in the Brazilian case on the day that the protesters took over the building of the Brazilian congress.

For the Mexican protest, activity follows a slow decreasing trend over time. In the Brazilian case, however, activity increases drastically midway through the observation period. This increase is followed by slow delay with occasional sharp increases. In both cases we find that organizations have a higher number of average tweets per day than individuals.

Interestingly, in the Brazilian case we find that organizations play an important role in posting protest-related content during the later months of the movement. While activity across all actor types increases drastically on June 15th, activity by individuals gradually returns to lower levels. Activity by organizations however maintains its increased level for months. One possible explanation for this is that the organizations behind some of the earliest protests that trigger the more large scale ones, are resilient and committed to long-term advocacy of the specific issues they care about. For example, the Movimento Passe Livre that was key to the first protests in 2013, has been advocating for free public transit since at least 2005. Hence the spike and dwindling in political mobilization in 2013, might have had no negative effect in their long-term commitment to their causes.

We compare activity with participation across time. Fig 2’s bottom pane shows the number of unique active (have posted a tweet) actors by category. In both cases studied, individuals make up the large majority of participants, despite producing less content, on average, per person. We also find, across cases, that organizations are more consistent (less prone to bursts) in their participation in the protest conversation over time. Together these results suggest that while the large majority of actors participating in protest-related communication on Twitter are individual actors, it is organizations who post the most content if we compare activity at the account level.

**Fig 3:**
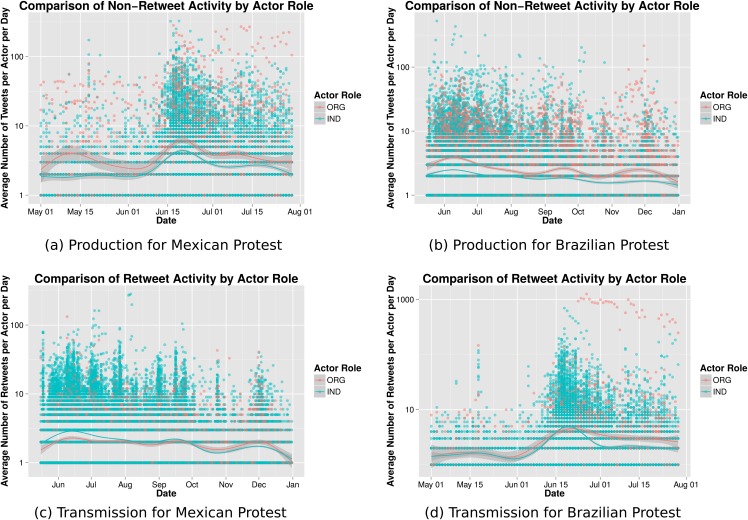
Content production and transmission by actor type for a sample of highly active users over time.

This initial look at the differing roles of organizations and individuals reveals some interesting patterns of behavior; it also raises additional questions. In particular, one interesting distinction we can make about social media posts is whether a post’s content was produced by the poster or whether the post is a retweet, indicating the poster is retransmitting another user’s content. In Fig 3 we compare these two very different types of engagement.

In the top panel of Fig 3 we show the average number of tweets per day by actor type, only considering content that is not a retweet, i.e. content that is produced by the posting actor. Likewise, in the bottom panel of Fig 3 we show the average number of retweets posted per day by actor type for each protest case. For both the Mexican and Brazilian protests we find that organizations produce more original, non-retweet, content than individuals across time, and that this difference remains consistent across time.

Fig 3 also demonstrates that in both events retweet activity varies across time. To begin it is individual actors who post more retweets per user, however, gradually over time organizations overtake individual actors in the average number of retweets posted per account. These results reinforce previous findings, suggesting that organizations play a vital role in sustaining protest-related communication on Twitter during the later stages of the movements.

### Interaction Dynamics

Social media platforms offer a valuable window into interpersonal interaction dynamics in disrupted settings such as protests [18, 25, 26]. Here we consider the overall pattern of interaction over time as measured through changes in the overall structure of the social network. Comparing the structure of the interaction network across time allows us to identify key points where interaction patterns are “abnormal” or different from other points in time.

**Fig 4:**
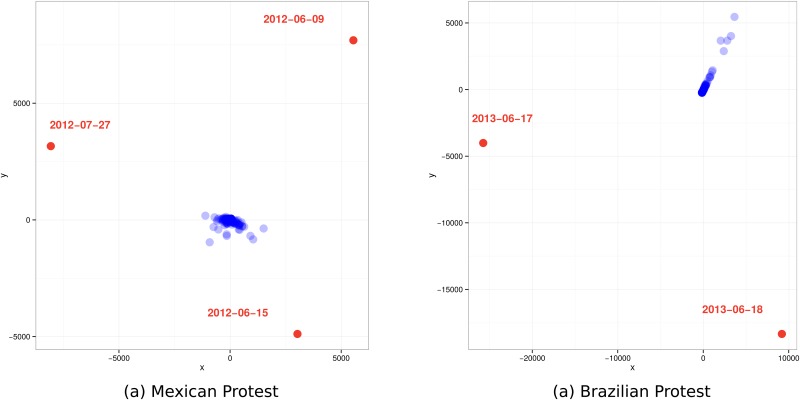
Interaction network structural dynamics. Each point represents a daily snapshot of the interaction network. The distance between points (two daily networks) represents their similarity—pairs closer in space are more similar.

We consider the sequence of daily snapshots of interaction. That is, we aggregate tweets into 24 hour windows and consider the sequence of these interaction networks. We want to characterize the changes that occur from one time point to the next. To do this we use a distance-based measure of network comparison. We calculate the Hamming distance between each pair of daily snapshots [27–29]. Hamming distance measures the number of edge (or social tie) changes that would need to be made in order to transform one network to be identical to a second network. This computation produces a matrix of similarity (or corresponding distances) scores for each pair of networks. We project the similarity matrix into 2-dimensional space using a standard multidimensional scaling, as seen in Fig 4.

We can then qualitatively compare the interaction networks over time. In this visualization each point represents a daily snapshot of the interaction network and the relative distance between points demonstrates their similarity—points close in space are more similar. It is important to note that the absolute distance between points has no concrete meaning, rather it is the relative distance that should be considered.

What we find is strikingly similar across the two cases. As Fig 4 shows, for the most part the daily interaction networks are very similar across time points, with a few notable exceptions. In both the Mexican and Brazilian cases there are a few time points that are radically different from all the rest. This implies that the same actors interact with each other consistently over time—in other words—the network has a “normal” state most of the time. However, on particular days the interaction patterns are unique.

Even more interesting is the fact that while these excursions exist, the social network returns to its normative state afterwards, demonstrating a cyclic pattern. In fact, if we consider the exogenous circumstances on these “outlier” interaction days we find that massive street protests tend to attract interaction among people who otherwise are not very likely to interact.

To further explore interaction dynamics during the two protest cases studied here, we look at the rates of particular directed interactions over time. There are four different types of pairwise interaction that can occur between our two actor categories of interest: (1) individuals can mention other individuals, (2) individuals can mention organizations, (3) organization can mention individuals or (4) organizations can mention other organizations.

To compare these types of interaction over time we again consider daily snapshots of the interaction network. We collapse actors by type and consider the rates of interaction within and between the two groups, controlling for the number of actors in each group. For those familiar with social network analysis, this is a blockmodel view of interaction based on actor covariates. We compare observed rates of interaction with a Poisson baseline given the number of actors and average activity to obtain a measure of how significant, or surprising, each kind of interaction is over time, which can be interpreted like a *p*-value.

**Fig 5:**
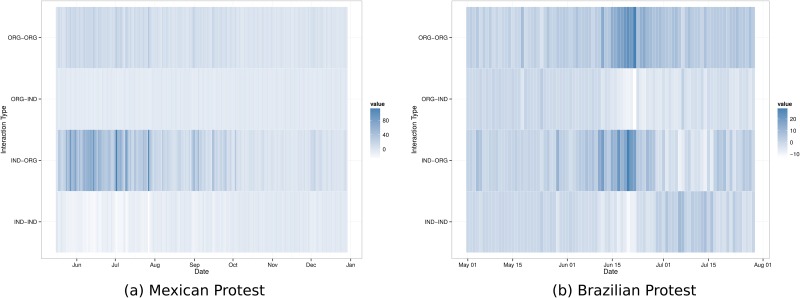
Directed online interaction between actor types over time for each social protest.

In Fig 5 we show these distinct paired interactions over time, cells are colored by the estimated *p*-value, darker cells are more surprising given the baseline. The goal in this analysis is to tease out different types of interaction that occur between groups and how these interactions change over time. For example, in the previous section we discussed the fact that in both protests organizations posted more retweets than individuals (on a per account basis) later during the protests’ life course. Are organizations more active in transmitting content posted by individuals or other organizations? How do these interaction dynamics change over time? These are the questions we address here.

It is evident from comparing Fig 5(a) and 5(b) that the Mexican and Brazilian cases differ in terms of which groups interact with each other over time. For the Mexican case, no one type of paired interaction stands out as more common than we might expect. Initially, we see slightly more activity directed from individuals towards organizations, but this lessens over time.

In the case of Brazil, however, we see a very striking pattern that corresponds with the drastic increase in online activity, and massive offline protests. In late June, pairwise activity shifts. We observe large increases in within-organization mentions, and in individuals mentioning organizations. This increase is coupled with a decrease in organizations mentioning individuals, and within individual interaction. These shifts seem to coincide with the institutional reactions to address some of the protestors’ concerns, including a televised speech by President Dilma Rousseff, followed by the measures taken by state governors and the National Chamber to directly address the issues such as taxes, corruption, and other themes raised by protests.

## Discussion and Conclusion

Social media increasingly play a role in distributed political protests, offering mechanisms for exchanging information and interacting with others. Scholars argue about the exact role that social media can or will play during these protests; however, it is clear from recent events that platforms like Twitter are heavily used. Some scholars point to the ability of these platforms to facilitate sharing, which has been identified as a key component of *connective action*, allowing individuals to personalize their participation and interaction with the protest.

Another aspect of this new frame is the distinct role of organizations. New forms of social media-enabled protests do not resemble traditional collective action in their reliance on the coordinating efforts of organizations. Instead, some scholars claim that connective action frames allow movements to survive with limited involvement of traditional institutions or organizations. In this work we contribute to our understanding of new forms of protest by exploring in detail the dynamic roles of both individuals and organizations over the course of two different protests—the #YoSoy132 student uprising in Mexico and the “bus rebellion” in Brazil.

Our analysis suggests that although individuals are prominent at the birth of these protests, demonstrating high levels of activity and participation, these individuals fade as the movement dwindles and organizations take over. During the later stages of both protests considered, it was organizations who produced content at high rates and had sustained levels of participation.

We also consider the dynamics of the interaction network, identifying key time points at which unique social structures manifest. In particular we find that unique social structures are coupled with major offline events such as prominent protests. Moreover, we find that individuals and organizations have distinct patterns of interaction with and across actor-type groups, suggesting a typical pattern of content produced by certain actor groups and propagated by others.
